# Environmental and lifestyle risk factors of breast cancer in Malta—a retrospective case-control study

**DOI:** 10.1186/s13167-016-0069-z

**Published:** 2016-09-20

**Authors:** John Paul Cauchi, Liberato Camilleri, Christian Scerri

**Affiliations:** 1 Department of Physiology and Biochemistry, University of Malta, Room 111, Msida, MSD 2080 Malta; 2 Statistics and Operations Research, Faculty of Science, University of Malta, Room 509, Maths and Physics Building, Msida, Malta; 3 Physiology and Biochemistry, Faculty of Medicine and Surgery, Biomedical Sciences Building, University of Malta, Msida, Malta

**Keywords:** Breast cancer, Environment, Lifestyle, Environmental health, Targeted prevention, Predictive medicine

## Abstract

**Aim and Background:**

Environmental exposures are known to play a role in the development of cancer, including breast cancer. There are known associations of breast cancer with environmental factors such as sunlight exposure, diet and exercise and alcohol consumption as well as physiological factors.

This study examines the prevalence of risk factors for breast cancer related to dietary intake, environment and lifestyle in the female population of Malta. Malta has had little research in this area, and therefore an exploratory study was carried out.

**Methods:**

A retrospective case-control design was applied. Two hundred cases and 403 controls were included. Both cases and controls were subjects without a known family history for breast cancer. Controls were age-matched to cases in an age-decade category roughly at a 2:1 ratio. Interviews were carried out face-to-face using a questionnaire designed by Maltese and Sicilian researchers, encompassing various factors including diet, lifestyle, physiological factors and medical history. Breast cancer risk was then analysed using both univariate and multivariate analyses. For factors having a metric scale, the Mann-Whitney test was used to compare mean scores, while for categorical factors, the chi-square test was used to compare percentages between the case and control groups. Statistical modelling was carried out using binary logistic regression to relate the likelihood of breast cancer to over 50 risk/protective factors analysed collectively.

**Results:**

Univariate analysis showed around 20 parameters of interest, 14 of which were statistically significant at a 0.05 level of significance. Logistic regression analysis identified 11 predictors of interest that were statistically significant. Tomato, coffee and canned meat consumption were associated with lower likelihood of breast cancer (OR = 0.988, 0.901, 0.892, respectively), whereas beans and cabbage consumption and low sodium salt were positively associated with breast cancer (OR = 1.045, 1.834, 1.028, respectively). Premenopausal status was associated with a lower risk of breast cancer compared to postmenopausal status (OR = 0.067). Not having experienced myocardial infarction was associated with lower odds of breast cancer (OR = 0.331). Increased height was also found to have a strong association with risk of breast cancer, with the odds of having breast cancer increasing for every centimetre increase in height (OR = 1.048). In terms of quantity, odds of having breast cancer were lower in those exposed to sunlight (OR = 0.891). The odds of having breast cancer were also lower in those not using the oral contraceptive pill (OR = 0.454).

**Conclusions:**

Various factors in this exploratory study were found to be associated with development of breast cancer. While causal conclusions cannot be made, tomato consumption is of particular interest, as these results corroborate findings found in other studies. A negative association of breast cancer with sunlight exposure and oral contraceptive pill use corroborates findings in other studies. Other associations with dietary intake can be explained by dietary changes. More robust studies in this area, including possible longitudinal studies, are warranted.

**Electronic supplementary material:**

The online version of this article (doi:10.1186/s13167-016-0069-z) contains supplementary material, which is available to authorized users.

## Background

Breast cancer is the most common type of cancer in women in Europe, with an estimated incidence of 494,100 cases in 2012 alone [1]. While known to have strong links with specific genetic mutations, particularly the *BRCA1* and *BRCA2* genes [2–4], little is known about possible environmental and lifestyle risk factors that could play a role in the aetiology of the disease. Increasingly, epigenetic factors are being recognised as playing a role in the development of cancers [5–10].

With a growing body of literature in this area of research, there are now known physiological, lifestyle and environmental risk factors that play a role in breast cancer. Alcohol consumption, obesity, physical activity and parity are some of the factors known to affect the risk of developing breast cancer [11–24]. While various studies on this topic have been carried out looking at specific parameters, few studies assess a wide variety of factors [22, 25]. No such study has been carried out in Malta in relation to breast cancer. Breast cancer is the most common cancer in Malta and also the most common cancer in females [26]. In Malta, breast screening is provided for free every 3 years to all women aged between 50 and 60 years.

This exploratory retrospective case-control study was carried out in Malta in order to investigate potential associations of environmental and lifestyle factors with breast cancer risk and highlight areas for future research.

## Methods

A retrospective case-control study design was adopted. NICE criteria were used to exclude individuals having a family history of breast cancer from this study [27]. Ethical approval was obtained for the study from the University Research Ethics Council of the University of Malta (UREC)^1^. Overall, 200 cases and 400 controls, matched for decadal age category (e.g. 40–49 years), were recruited in this study at a ratio of two controls per case. Cases were recruited from clients who had attended the Breast Surgical Units of Malta’s central hospital since 2009, as well as current clients from the Molecular Genetics Clinic at the same facility. These 200 recruits were obtained in decadal bands corresponding to a ratio of decadal age of the total number of patients diagnosed with breast cancer since January 2009. Controls were recruited at random using the Malta April 2015 electoral register. A case was defined as “*a woman without a family history of breast cancer according to the NICE criteria who had breast cancer since 2009*” and a control was defined as “*a woman without breast cancer who has not had a history of breast cancer*” [27].

Contact details of potential cases were obtained from the 2009–2013 Malta breast clinic register. Cases were first contacted via telephone by nurses working at the breast surgical clinic. This was expected to increase participation rate as breast cancer patients would have interacted with and developed familiarity with nursing staff during previous visits to the clinic. The nurses also received training in telephone surveying skills following a script as a guideline. If they voluntarily agreed to participate in the study, participants’ information was passed on to the research team. These potential cases were then assessed by the research team engaged in this study for eligibility according to the NICE criteria prior to recruitment in the study. Controls were contacted first through a letter containing information about study and subsequently followed by a telephone call by members of the research team to offer further explanation, confirm eligibility and clarify any queries. Controls were recruited at a 2:1 ratio (Table 1). In all situations, anonymisation of data was assured, and participants were informed that they could drop out of the study at any time without providing a reason for doing so.

**Table 1:** Population distribution of cases and controls by age category

		Group		Total
		Control	Case	
Age category	20–29	2	1	3
	30–39	15	7	22
	40–49	62	30	92
	50–59	134	67	201
	60–69	102	51	153
	70–79	88	44	132
	Total	403	200	603

Following an extensive literature review on factors that are known or suspected to have a link with breast cancer, a questionnaire on dietary and lifestyle behaviour that encompassed wide variety of parameters was designed, [11–17, 26, 28–37]. It broadly examined demographics, physiology, lifestyle, diet (in the form of a food frequency questionnaire), work exposure and medication [38]. Face-to-face interviews were carried out by female interviewers from the research team who were trained to put interviewees at ease during questioning. Weight and height were also measured objectively, using a portable weighing scale and a measuring tape during the interview. Additionally, the volumetric measurements in the dietary recall section of the questionnaire were accompanied by pictures showing a standard size corresponding to the amount. This was done in an attempt to reduce portion size recall bias as well as to ensure standardisation of sizes between interviewers. Following the completion of the interviews, a back-check of 10 % of the samples was conducted to verify data integrity.

### Measurements

The following subsection will outline the measurement criteria for the items that resulted as statistically significant in their effect following multinomial regression.

#### Dietary

The standard unit for tomatoes was one medium tomato (≈110 g); that for beans and cabbages was one cup (28 g); that for canned meat was one tin (≈70 g); that for low sodium salt was one teaspoon; and that for coffee consumption was one cup (200 ml). A large number of other food items were measured in the food frequency questionnaire but as they were not found to have a significant impact in the multinomial regression, they were not included in further analysis and are listed in Additional files 1 and 2. In all cases, the values provided were input as consumption per week and then calculated as consumption per month.

#### Lifestyle and health

Interviewees were asked about lifetime use, frequency and type of oral contraceptive pill (OCP) used. However, as few respondents were able to elaborate on the type of OCP used, this variable was excluded from further analysis. In the case of sun exposure, the question asked looked at exposure during the summer months in order to assess the likelihood of sunbathing, specifically when they were 10–29 years of age. This age bracket was specified because it was assumed that exposure would be highest at that age, and it is also indicative of an individual’s sunlight exposure at its greatest intensity, during summer:

*Between the age of 10 and 29 did you use to spend at least an hour per day exposed to the sun during the summer months (July and September)? If yes, how many hours per day on average?*

The number of hours of exposure was then estimated.

Interviewees were also asked about their menopausal status, their age at onset of menopause and whether they had ever been diagnosed with an acute myocardial infarction by a medical professional.

#### Work exposure

During the interview, questions relating to chemical exposures that are suspected to increase the risk of developing breast cancer in certain studies were asked. These included solvents, pesticide use, use of cosmetics (with parabens known to trigger cancers in vitro), animal feed and medication (often containing hormones with unknown effect on humans) and industrial chemical exposure [39–42]. Those exposed to any of the above were considered to have *work exposure risk*.

### Interviews and data input

The research was subsequently carried out face-to-face by trained interviewers at a location and time convenient for the interviewee, which in the vast majority of the cases was at the home of the interviewee in a private room without other family members present. Interviews were carried out in English or Maltese; questionnaires were available in both languages. The questions were asked, and the answers inputted by the interviewer. Prior to commencing all interviews, consent was obtained from the subject. The interview questionnaire on average lasted around 35 min. Data was later inputted into Microsoft Excel, cleaned and analysed using SPSS v22.0.

## Results

Univariate logistic regression models were used to assess the association between the likelihood of breast cancer and any one of the predictors related to physiological characteristics, lifestyle, work, diet, illness and medication. Besides descriptive statistics, the table below displays the odds ratio for each predictor and its corresponding 95 % confidence intervals. A predictor with a *p* value less than the 0.05 level of significance was taken as significant, implying that the odds ratio is significantly different from 1 (95 % confidence for odds ratio excludes 1).

The distribution of each variable was assessed, and it was found that all continuous variables did not follow normal distribution. Univariate analysis was then carried out for every variable, using the non-parametric Mann-Whitney test for differences between continuous variables and chi-square test for Association for categorical variables. Following univariate analyses, all variables with a *p* value of less than 0.1 were included in a multivariate logistic regression model. The Akaike Information Criterion Index (AIC) and the Pseudo R-Square values (focusing on Nagelkerke value) were used in deciding the best parsimonious model selection. Variables that had considerable missing data were not included in the model. Using a forward stepwise model, odds ratios (OR) of the most significant variables in the model were obtained. The parsimonious multinomial logistic model (Table 3) therefore provided an indication as to which variables would prove more interesting for future research.

In Table 2, “*r*” is the effect size which is equal to the z-score divided by the square root of the sample size. The effect size *r* is an alternative representation of the *p* value because both the effect size *r* and the *p* value depend on the z-score.

**Table 2:** Mann-Whitney analysis and odds ratio assessing the association between the likelihood of breast cancer and any one of the predictors related to physiological characteristics, lifestyle, work, diet, illness and medication

	Mann-Whitney test						Binomial logistic regression
	Cases	Control	*U*	*Z*	*p* value	*r*	Odds ratio
	*n*(%)/mean	*n*(%)/mean					(95 % conf. int.)
Lifestyle							
School leaving age	200 (15.35)	401 (15.75)	36191	−1.947	0.044	0.08	0.931 (0.86, 0.99)
Moisturiser amount (ml/week)	200 (10.78)	403 (15.57)	33958	−3.261	0.001	0.133	0.582 (0.39, 0.87)
Makeup/moisturiser use (yes = 1)	200 (0.73)	401 (0.82)	36373	−2.643	0.008	0.108	0.986 (0.98, 0.99)
Sunlight exposure age 10–29 (yes = 1)	200 (0.75)	403 (0.82)	37525	−1.974	0.048	0.081	1.507 (1.00, 2.27)
Work							
Ever worked (yes = 1)	200 (0.79)	401 (0.86)	37179	−2.285	−0.022	0.094	0.598 (0.38, 0.93)
Diet							
Tomatoes (no./month)	200 (25.7)	403 (31.9)	33363	−3.489	<0.001	0.143	0.992 (0.986, 0.999)
Beans (cups/month)	200 (5.62)	403 (3.80)	35120	−2.632	0.008	0.108	1.038 (1.01, 1.06)
Cabbage (cups/month)	200 (1.378)	403 (0.07)	30963	−9.361	<0.001	0.382	1.732 (1.36, 2.22)
Chinese food (meals/month)	200 (0.19)	403 (0.32)	37428	−2.314	0.021	0.095	0.837 (0.66, 1.06)
Soya exposure (cups per month)	200 (1.25)	403 (1.22)	35594	−2.915	0.004	0.119	1.001 (0.97, 1.03)
Soft drink (33 cl cans/month)	200 (4.45)	403 (8.79)	34911	−3.085	0.002	0.126	0.982 (0.97, 1.00)
Illness							
Myocardial episodes (yes = 1)	200 (0.08)	403 (0.03)	38678	−2.174	0.03	0.089	2.253 (1.07, 4.77)
Medication							
Diuretics (yes = 1)	200 (0.19)	403 (0.12)	37545	−2.281	0.023	0.093	1.719 (1.08, 2.75)
Anti-hypertensives (yes = 1)	200 (0.29)	403 (0.17)	35715	−3.233	0.001	0.132	1.929 (1.29, 2.88)
ACE inhibitors (yes = 1)	200 (0.12)	403 (0.07)	38364	–1.96	0.05	0.08	1.759 (1.00, 3.11)

The following predictors were found not to be statistically significant. *Physiological characteristics*: height, weight, BMI, menarchal age, parity, menopausal age, menopausal status, OCP use, HRT use; *Lifestyle*: exercise, exercise, alcohol status, alcohol lifetime exposure, tobacco, tobacco lifetime exposure, second-hand smoker, makeup amount, vegetable washing, sun exposure; *Work*: work exposure risk; *Diet*: carrots, spinach, artificial sweetener, low sodium salt, dried soup, soysauce, chips, canned meat, illness, diabetes, liver failure episodes, hypothyroidism; *Medication*: metformin, calcium-channel blockers

The goal of this research study is to assess the impact of the predictors collectively on the likelihood of breast cancer. It is well known that a significant lone predictor could be rendered unimportant in the presence of other predictors, while an insignificant lone predictor could be rendered important if included with other predictors. In other words, the appropriateness of a predictor in a model often depends on what other predictors are included with it. The parsimonious binary logistic regression model using a forward stepwise procedure was utilised.

The parsimonious model relating the likelihood of breast cancer (case vs. control) to a number of risk/protective factors identifies 11 significant predictors, which include the consumption of tomatoes, beans, cabbage, low sodium salt consumption, canned meat, coffee consumption, use of oral contraceptive pills, menopausal status, incidence of myocardial infarction, exercise, height and exposure to sunlight. This parsimonious model explains 31.7 % of the total variance in the breast cancer outcomes (Nagelkerke).

The Pseudo R-square value is rather low indicating that there are predictors (not included in this study) that explains the remaining 68.3 % of the total variance. One of these predictors is undoubtedly age, where it has been found in literature that an increase in age increases the prevalence of breast cancer. This variable was excluded in this logistic regression analysis since cases and controls had been matched according to age category.

### Interpretation of results

The 11 significant predictors shown in Table 3 will now be interpreted based on their category and their relationship to the response variable (likelihood of breast cancer).

**Table 3:** Parsimonious logistic regression model

Group	*B*	Std. error	*p* value	Odds ratio	95 % conf. int. for odds ratio	
Ref. cat = control					Lower bound	Upper bound
Intercept	−5.651	2.116	.008			
Tomatoes	−0.012	.004	.002	.988	.981	.996
Cabbage	0.607	.133	.000	1.834	1.414	2.380
Beans	0.044	.014	.002	1.045	1.016	1.075
Low sodium salt	0.028	.013	.036	1.028	1.002	1.055
Canned meat	−0.115	.046	.013	.892	.815	.976
American coffee	−0.104	.053	.048	.901	.813	.999
No OCP Uses OCP	−0.790 0	.338	.019	.454	.234	.879
Premenopausal Perimenopausal Postmenopausal	−2.704 −0.483 0	.501 .285	.000 .090	.067 .617	.025 .353	.179 1.077
No MI Had MI	−1.107 0	.421 .	.009 .	.331 .	.145 .	.755 .
Height (cm)	0.047	.014	.000	1.048	1.021	1.077
Sun exposure	−.115	.049	.020	.891	.809	.982

#### Dietary intake

The parsimonious model identifies six significant dietary predictors, which include tomatoes, beans, cabbage, canned meat and coffee consumption. For every medium-sized tomato consumed per month, for every tin (approx. 70 g) of processed meat consumed per month and for every cup of coffee consumed per month, the odds of having breast cancer decreases by 1.2, 10.8 and 9.9 %, respectively. These three diet predictors were found to be significant protective factors.

On the other hand, for every cup (28 g) of fava beans consumed per month, for every cup of cabbage consumed per month and for every teaspoon of low sodium salt consumed, the odds of having breast cancer increases by 4.5, 83.4 and 2.8 %, respectively. These three diet predictors were found to be significant risk factors.

#### Physiological factors

The parsimonious model also identifies three significant physiological factors, which include history of myocardial infarction, menopausal status and height of the female. For subjects with no history of heart attack, the odds of having breast cancer is 66.9 % lower compared to subjects who have reported at least one episode of heart attack. For premenopausal and perimenopausal women, the odds of having breast cancer are, respectively, are 93.3 and 38.3 % lower compared to postmenopausal women. On the other hand, for every centimetre increase in height, the odds of having breast cancer increases by 4.8 %. Hence, tall postmenopausal women with reported episodes of cardiac problems are more likely to develop breast cancer than short premenopausal women who have no history of heart problems.

#### Lifestyle

The parsimonious model identifies two significant predictors of breast cancer, which include exposure to summer sunlight and use of the oral contraceptive pill. For every additional hour of sunlight exposure during summer, the odds of having breast cancer decreases by 10.9 %. For women not taking oral contraceptive pills, the odds of having breast cancer is 54.6 % lower than that for women taking oral contraceptive pills.

#### Work

As described above, work exposure risk was measured in this study. However, it did not appear as significant in the univariate analysis, and work exposure also was not significant in the parsimonious model.

## Discussion

### Diet and dietary change

The results above show that tomatoes and canned meat consumption seem to decrease the odds of developing breast cancer. On the contrary, beans, low sodium salt and cabbage consumption seem to increase the odds of developing cancer.

There are a number of confounding issues when identifying food items as risk or protective factors of breast cancer.

- Other food items are being consumed with the item analysed, which are contributing to the risk or protective factors of breast cancer; however, these items are not included in the study.
- Dietary changes followed by subjects after being diagnosed with breast cancer.
- The list of item being analysed may be too generic—for instance, the study makes reference to fava beans; however, the subject may mix it up with other types of beans that may have a different effect on breast cancer.

The onset of a health condition is very often followed by a change in the food diet. For instance, concerns over high blood pressure levels may lead subjects to change regular salt intake with low sodium salts. Similarly, patients diagnosed with breast cancer may change their food diet to a healthier one. Beans and cabbages are often considered to be part of a healthy diet and normally are not associated with adverse effect on breast cancer. Our study shows that cabbages, beans and low salt intake are more likely to increase the risk of breast cancer, while canned meat and American coffee are more likely to protect against cancer. These anomalous results may be attributed to the fact that subjects diagnosed with breast cancer tend to change their food diet to a healthier one. Hence, there are larger proportions in the case group eating cabbages, beans and low salt and larger proportions in the control group taking canned meat and American coffee. At least one study has found that in premenopausal women, regular coffee drinking appears to be linked with a reduced risk of the disease [43].

In the case of tomato consumption, our result complements other findings in literature, which identify tomato consumption as a protective factor against breast cancer. Other studies [44, 45] found that lycopene compounds have antioxidant effects that protect against free-radical damage which is associated with an increased risk of developing cancer. In fact, tomatoes are known to be particularly rich in lycopene. Tomatoes are frequently consumed in Malta and other Mediterranean countries, and subject recall bias is less likely when they quantify tomato consumption per week.

### Lifestyle

Exposure to sunlight seems to be associated with a decrease in the risk of breast cancer. This corroborates findings found in other studies [24, 46]. It is highly possible that in both controls and cases, subjective recall bias influences the accuracy of estimated exposure to sunlight. Most people find it difficult to recall the exact period of time they spent directly exposed to sunlight, and some subjects might have different ideas as to what qualifies as “direct exposure” even if the interviewer made it as clear as possible. The time of day of sunlight exposure is another confounding factor since sunlight exposure is more intense during certain hours of the day, which may actually counteract any benefit.

Our study shows that exposure to sunlight has a positive effect on the prevention of breast cancer. This finding is also corroborated in other scientific analyses of this nature [24, 46, 47]. Though prolonged sunlight exposure damages the skin, it has been found that it reduces the risk of breast cancer—possibly due to vitamin D production and its role in breast cell growth. Therefore, one cannot discount these findings as mere artefacts or results of recall bias.

#### Oral contraceptive pill use

As reported in the results section, the odds of having breast cancer *is* 54.6 % *lower* in subjects not taking oral contraceptive pills compared to those who take oral contraceptive pills. This is quite a large effect, and corroborates established scientific evidence that oral contraceptive pill use increases breast cancer [48].

### Physiological factors

The three physiological factors emerging as highly significant were height, a history of myocardial infarction (heart attack) and menopausal status.

#### Height

Height came as a much unexpected factor in this analysis, appearing as significant in virtually every model run in this study. The model selected showed a 4.8 % increase in the odds of having breast cancer for every centimetre increase in height (*p* value <0.001; CI = 1.021 cm, 1.077 cm). Large-scale studies carried out elsewhere which have examined height as a continuous variable also found such an association [49]. One possible explanation could be that taller individuals have a greater number of cells, and this would statistically lead to a greater risk of a cell going awry and turning cancerous; however, few studies have been carried out linking height to cancer, and further research is necessary prior to making any conclusions. There seems to be no association between breast size and height [50].

The height effect on the prevalence of breast cancer is doubtful due to a number of confounding factors. Breast tissue density was not taken into account, and neither was the type of breast cancer. Both these parameters are known to be far more associated with breast cancer risk than height. Although these two effects could not be included in this study, the height effect as risk factor of breast cancer cannot be entirely disregarded.

#### Myocardial infarction

For women with no history of heart attacks, the odds of having breast cancer was 66.9 % lower than their counterparts with a family history. This difference cannot be attributed to age differences between cases and controls, as the study cases and controls were age-matched. Literature shows that living a healthy lifestyle not only reduces the risk of cardiovascular ailments but also lowers the risk of cancers, including breast cancer.

#### Menopausal status (categorical)

Postmenopausal women are more likely to develop breast cancer than their younger counterparts. Logistic regression analysis shows that for premenopausal women and perimenopausal women, the odds of having breast cancer rather than not having breast cancer are, respectively, 93.3 and 38.3 % lower compared to those for postmenopausal women. Table 4 outlines the menopausal status according to age category and sorted as case-control.

**Table 4:** Menopausal status sorted according to age and case-control status

			Menopausal status		
			Premenopausal	Perimenopausal	Postmenopausal
Control	Age category	20–29	100.0 %		
		30–39	93.3 %		6.7 %
		40–49	83.6 %	11.5 %	4.9 %
		50–59	13.5 %	36.1 %	50.4 %
		60–69	2.0 %	6.9 %	91.2 %
		70–79			100.0 %
	Total		21.7 %	15.5 %	62.8 %
Case	Age category	20–29	100.0 %		
		30–39	42.9 %	42.9 %	14.3 %
		40–49	6.7 %	36.7 %	56.7 %
		50–59		17.9 %	82.1 %
		60–69		4.0 %	96.0 %
		70–79			100.0 %
	Total		3.0 %	14.1 %	82.9 %

It is well known that age is a strong risk factor of breast cancer and postmenopausal women tend to be older than premenopausal and perimenopausal women. Another explanation, however, can be attributed to the fact that women who start menopause at a later age tend to have had greater exposure to oestrogens in their lifetime, and therefore are at an increased risk of developing breast cancer.

Table 4 shows a smaller proportion of women in the control group (4.9 %) who were in a postmenopausal state between the age of 40 and 49 years, compared to women in the case group (56.7 %). On the other hand, there is a larger proportion of women in the control group (13.5 %) who were in a premenopausal state between the age of 50 and 59 years, compared to women in the case group (0.0 %). These figures show that women who commence their postmenopausal state at a young age are more at risk of breast cancer than women who have a delayed menopause.

### Limitations—bias and confounding

Retrospective case-control studies are prone to recall bias and cannot be used to establish causality. The main problem with case-control studies and dietary questionnaires is that recall bias tends to be unavoidable. In this study for example, people were not recruited upon diagnosis, and therefore, one could not assess diet prior to diagnosis. It is known the dietary changes do occur more often in those who have suffered a life-threatening pathology such as breast cancer. This was reflected in our findings in the study, as shall be discussed below.

Recall could not be avoided in this questionnaire, as people in general do not really know the amount of an item they typically consume in a week. Additionally, case subjects in the study might be more cautious as to which items they eat and consume in light of their pathology, and therefore, the reporting from cases is probably more accurate in this regard. In the same way, however, the cases might not be so willing to report consumption of items they deem as “harmful to health” so as to avoid embarrassment. One can therefore never truly know how often an item is consumed in a case-control study.

Recall bias also becomes a problem in the older strata of the population where due to a decrease in cognitive function accurate recall might be a problem. This study included a large number of women defined as “elderly” that might or might not have been suffering from memory impairment due to ageing.

In an attempt to address the problem of recall bias, questions asked were limited to “*how much of item X do you consume per week*” and then added up to a monthly figure. Additionally, images of containers were shown in an attempt to introduce standardisation of quantity consumed across subjects. These attempts probably went some way at addressing the issue of quantity standardisation and gave a picture as to a “typical” diet. However, recall bias remains a factor in this study.

Confounding factors are also a significant problem in retrospective case-control studies such as the one carried out here, as in most situations there are various confounding factors that cannot be catered for. One example is work exposure. While questions were asked as to possible exposures, the subjects who provided an answer were too few for statistical significance to be reached. This could also be true for various food factors, where a complete dietary analysis was impossible to carry out given timeframes and available funds.

Another major limitation in the study is that breast cancer was not categorised by individual subtype. Different cancers might have different aetiologies and different risk factors contributing to their possible emergence. However, this data was not readily available and while it would have been obtainable, it was not deemed central for this study, which was conducted as an exploratory exercise. Additionally, the relatively small cohort of people involved in the study would have made any statistically significant results hard to achieve if one were to look at sub-categories.

However, despite the obvious limitations, these results can form the basis of further studies focusing on the factors that were indicated as showing a significant effect on the occurrence of breast cancer in general. The odds ratios discussed in the results section should therefore be viewed with caution, given the magnitude of recall and confounding bias. One cannot view the results in a quantitative matter but rather as possible indicators for future studies in this field. On the other hand, a number of factors found to be associated with breast cancer are actually substantiating other published work.

## Conclusions

The findings of this study indicate diet, lifestyle and physiological factors as possible factors that play a role in the risk of developing breast cancer. This particular study, however, suffered from problems intrinsic to case-control studies, namely recall bias and confounding effects.

### Expert recommendations and outlook

The exploratory nature of the study carried out cannot imply causality, but it can have public health value in indicating what people should seek or avoid in their daily lives in terms of possible exposures that increase the risk for breast cancer.

Given the above results, it is suggested that in the future, cohort studies can be carried out over a longer-time period so as to further establish the link between environmental and lifestyle risk factors and their role in breast cancer occurrence. The findings here warrant a further investigation into the area, if possible, using a larger population sample and a refined, concise questionnaire so as to gather more accurate data. Additionally, sub-categorisation of breast cancer types should be included in future studies, reflecting the different possible aetiologies relating to different categories of breast cancer. In the general public, the information above can be informative, especially when it comes to dietary adaptation. This study could prove useful in pointing the way to a more integrative approach in personalised medicine [51].

## Abbreviations

MI, myocardial infarction; OR, odds ratio; OCP, oral contraceptive pill

## Additional files

Additional file 1: Questionnaire. (DOCX 64 kb)

Additional file 2: List of variables analysed. (DOCX 14 kb)

## Footnotes

1 UREC Reference number for the study was MD63/2013, obtained in March 2014.
