# Supplementary material for: Environmental and lifestyle risk factors of breast cancer in Malta—a retrospective case-control study
Source: EPMA J. 2016 Sep 20;7(1):20. doi: 10.1186/s13167-016-0069-z (PMC5029064; doi:10.1186/s13167-016-0069-z)
Supplement: Supplementary file 1 — Questionnaire. (DOCX 64 kb) [file 13167_2016_69_MOESM1_ESM.docx]

Additional file 1 - Questionnaire

# Section 1: Interviewee Details

Q1. Reference Number: MT_________

Q2. How old are you? ________ years

# Section 2: Location

Q3. Can you please give me your address?

○ Refused

○ Lives Abroad/ Has until very recently (6 months) lived abroad

Home number/name: ______________________________

Street name: ______________________________

Post code: ______________________________

Locality: ______________________________

Q4. How many years have you been living in this location?

*Round up or down to the nearest year*

________ years

Q5. Where did you live prior to your current address? *Home number not required*

○ I have always lived at the same address – **GO TO SECTION 4 (pg2)**

○ Refused

○ Lived Abroad

Street name: ______________________________

Post code: ______________________________

Locality: ______________________________

**ASK Q6 IF THE RESPONDENT HAS LIVED IN A PREVIOUS LOCATION**

Q6. For how many years did you live in this location?

*Round up or down to the nearest year*

________ years

# Section 3: Physiological Section

Q7. Measurements to get BMI

| Weight (in kg) *please ask for a scale* | Height (in metres) *Please use the inch tape provided* |
| --- | --- |
|  |  |

Q8. At what age did you have your first period?

____________ years

Q9. How many children do you have?

____________ children

○ Never had children – GO TO Q12 *(for inputting write in ‘0’)*

Q10. At what age did you have your first child?

____________ years

Q11. If you breast-fed, for how long in total?

*Total amount of breastfeeding time in months*

____________ months *(for inputting write in ‘0’)*

○ Never breastfed

Q12. Are you Menopausal?

1. Pre-menopausal **- GO TO Q14**
2. Peri-menopausal (currently experiencing menopause)
3. Post-menopausal
4. No info given **- GO TO Q14**

Q13. At what age did Menopause start?

____________ years

Q14. Have you taken HRT? How long did you take it for?

*Hormone Replacement Therapy*

1. Never
2. More than 5 years duration
3. Less than 5 years duration
4. Currently using HRT

Q15. Do you use oral contraceptive Pills?

1. Yes **– GO TO Q16**
2. No – **GO TO CLOSURE SECTION**

Q16. Contraceptive Pills usage:

*Ask respondent to try and recall OCP used, From – To year*

| *From* | *To* | *OCP Used* |  |
| --- | --- | --- | --- |
|  |  |  | ○ Don’t remember |
|  |  |  | ○ Don’t remember |
|  |  |  | ○ Don’t remember |
|  |  |  | ○ Don’t remember |
|  |  |  | ○ Don’t remember |

# Section 4: Occupational History Section

Q17. At what age did you leave school? *(meaning formal full time education)*

________ years

Q18. Did you attend further education?

1. Yes
2. No

Q19. What level of education have you attained so far?

1. Primary
2. Secondary
3. Sixth Form
4. Diploma
5. Undergraduate Degree
6. Postgrad Degree (Masters)
7. Academic Fellowship (PHD)

Q20. Have you ever worked?

1. Yes - **GO TO SECTION 4B WORK EXPOSURE SECTION**
2. No - **GO TO SECTION 5: LIFESTYLE SECTION (pg27)**

# Section 4Bi: Work – Exposure Section

**Please list all the work you have done. At least for 6 months starting from your last regular work.**

*INTERVIEWER: Please specify whether the work actually involved exposure to the substances (eg. A manager of Delimara PS may have had no contact with solvents, etc.)*

**IF ONLY ONE JOB WAS CARRIED OUT FILL IN THIS SECTION ONCE; IF MORE THAN ONE PREVIOUS JOB RETAINED FOR OVER 6 MONTHS FILL IN AS MANY SECTIONS AS REQUIRED (UP TO THE LAST 4 SECTIONS HAVE BEEN PROVIDED)**

1. **Job Category (write in) _____________________________________________**

*Here it is up to the interviewer to decide the category that is “most fitting”. For eg. A cleaner would be an “operator” as she would be exposed to cleaning agents, etc.*

1. Administrative
2. Operator
3. Supervisor / middle management
4. Manager
5. Director
6. **FROM** (*year & month) ____________________* **TO** (*year & month) ____________________*

*(for inputting purposes always use the first of the month)*

1. **At this workplace was there use of:**
2. Solvents – **ASK SECTION 4Ci: SOLVENTS EXPOSURE (pg 4)**
3. Pesticides/Herbicides – **ASK SECTION 4Di: PESTICIDES/HERBICIDES (pg 5)**
4. Chemical Fumes or Gases – **ASK SECTION 4Ei: CHEMICAL FUMES/GASES (pg 6)**
5. Animal Feed – **ASK SECTION 4Fi: ANIMAL FEED (pg 7)**
6. Pharmaceutical/Cosmetic Products – **ASK SECTION 4Gi: PHARMACEUTICAL/COSMETIC**

**PRODUCTS (pg 8)**

1. None of the above - GO TO NEXT SECTION (pg 9), LIST THE PREVIOUS

JOB, IF NO OTHER JOB GO TO SECTION 5 (pg 27)

# Section 4Ci: Solvent Exposure

1. Brief Description of Task (*Insert keywords ONLY such as “mixing” or “painting”, include type of solvent if known)*

*_____________________________________________________________________*

*_____________________________________________________________________*

*_____________________________________________________________________*

1. Approximately how many hours per day involved contact with the solvent?

__________ hours

1. Approximately how many days involved contact with the solvent? And how frequent was this?

| Number of days:  _____________________ | Frequency:   1. Weekly 2. Monthly 3. Yearly |
| --- | --- |

1. How much of the solvent/mixture was used per day?
2. _____ spoonful (10ml per spoonful)
3. _____ cup (200ml per cup)
4. I don’t remember
5. At this work did you ever:
6. Have regular headaches
7. Experience dizzy spells/ fainting
8. Become unconscious (excluding head injury)
9. None of the above *(for online input leave empty)*

# Section 4Di: Pesticide/Herbicide Exposure

1. Brief Description of Task (*Insert keywords ONLY such as “vaporisation” or “pouring”, “mix”, include type of pesticide if known)*

*_____________________________________________________________________*

*_____________________________________________________________________*

*_____________________________________________________________________*

1. Approximately how many *days* per year involved contact with the pesticide/herbicide?

__________days

1. How much of the *diluted* pesticide was used every time?
2. 1 cup (200ml)
3. 2 to 5 cups (400 to 1000ml)
4. 6 to 9 cups (1200 to 2000ml)
5. 10 cups or more (>2000ml – 2lts)
6. I don’t remember
7. Have you ever been treated by a doctor for poisoning by these chemicals?
8. Yes
9. No

# Section 4Ei: Chemicals/Gases Exposure

1. Brief Description of Task (*Insert keywords ONLY such as “Laboratory – quality assurance” or “Industries - Chemical”, “Research”, include type of chemical/gas if known)*

*_____________________________________________________________________*

*_____________________________________________________________________*

*_____________________________________________________________________*

1. On average how many hours per day involved contact with the chemicals/gases?

__________ hours

1. Approximately how many days per week involved contact with the chemicals/gases?

○ 1 ○ 2 ○ 3 ○ 4 ○ 5 ○ 6 ○ 7

1. How much of the chemical/gases was used per day?

1. Less than 50ml

2. 51 to 500ml

3. 501 – 2000ml

4. More than 2000ml (2lts+)

5. I don’t remember

5. At this work did you ever:

1. Have regular headaches
2. Experience dizzy spells/fainting
3. Become unconscious (excluding head injury)
4. None of the above *(for online input leave empty)*

# Section 4Fi: Animal Feed Exposure

1. Brief Description of Task (*Insert keywords ONLY such as “Stable Farm Cows” or “Pigs” , “Chicken steroid-enriched food” include type of food used)*

*_____________________________________________________________________*

*_____________________________________________________________________*

*_____________________________________________________________________*

1. On average how many hours per day involved contact with animal feed?

__________ hours

3. Approximately how many days per week involved contact with the animal feed?

○ 1 ○ 2 ○ 3 ○ 4 ○ 5 ○ 6 ○ 7

4. How much of the animal feed was used per day?

1. Less than 15 kg
2. 16 to 30 kg
3. 31 to 50 kg
4. More than 50kg
5. I don’t remember

# Section 4Gi: Cosmetics and Pharmaceuticals Exposure

1. Brief Description of Task (*Insert keywords ONLY such as “mixing” or “vaporisation”)*

*_____________________________________________________________________*

*_____________________________________________________________________*

*_____________________________________________________________________*

1. Approximately how many hours per day involved contact with the product?

__________hours

1. How much of the cosmetic/pharmaceutical product was handled per day? (note: ONLY lab work)
2. Less than 5ml (1 teaspoon eg.)
3. 5 to 10 ml
4. 11 to 20 ml
5. More than 20 ml
6. I don’t remember
7. Not applicable (did not work in Lab)
8. Do you use a microfilter during work?
9. Yes
10. No

# Section 4Bii: Work – Exposure Section

**Please list all the work you have done. At least for 6 months starting from your last regular work.**

*INTERVIEWER: Please specify whether the work actually involved exposure to the substances (eg. A manager of Delimara PS may have had no contact with solvents, etc.)*

**IF ONLY ONE JOB WAS CARRIED OUT FILL IN THIS SECTION ONCE; IF MORE THAN ONE PREVIOUS JOB FILL IN AS MANY AS REQUIRED UP TO THE LAST 4 JOBS**

1. **Job Category (write in) _____________________________________________**

*Here it is up to the interviewer to decide the category that is “most fitting”. For eg. A cleaner would be an “operator” as she would be exposed to cleaning agents, etc.*

1. Administrative
2. Operator
3. Supervisor / middle management
4. Manager
5. Director
6. **FROM** (*year & month) ____________________* **TO** (*year & month) ____________________*
7. **At this workplace was there use of:**
8. Solvents – **ASK SECTION 4Cii: SOLVENTS EXPOSURE (pg10)**
9. Pesticides/Herbicides – **ASK SECTION 4Dii: PESTICIDES/HERBICIDES (pg11)**
10. Chemical Fumes or Gases – **ASK SECTION 4Eii: CHEMICAL FUMES/GASES (pg12)**
11. Animal Feed – **ASK SECTION 4Fii: ANIMAL FEED (pg13)**
12. Pharmaceutical/Cosmetic Products – **ASK SECTION 4Gii: PHARMACEUTICAL/COSMETIC**

**PRODUCTS (pg 14)**

1. None of the above - GO TO NEXT SECTION (pg15), LIST THE PREVIOUS

JOB, IF NO OTHER JOB GO TO SECTION 5 (pg27)

# Section 4Cii: Solvent Exposure

1. Brief Description of Task (*Insert keywords ONLY such as “mixing” or “painting”, include type of solvent if known)*

*_____________________________________________________________________*

*_____________________________________________________________________*

*_____________________________________________________________________*

1. Approximately how many hours per day involved contact with the solvent?

__________ hours

1. Approximately how many days involved contact with the solvent? And how frequent was this?

| Number of days:  _____________________ | Frequency:   1. Weekly 2. Monthly 3. Yearly |
| --- | --- |

1. How much of the solvent/mixture was used per day?
2. ____ spoonful (10ml per spoonful)
3. ____ cup (200ml per cup)
4. I don’t remember
5. At this work did you ever:
6. Have regular headaches
7. Experience dizzy spells/fainting
8. Become unconscious (excluding head injury)
9. None of the above

# Section 4Dii: Pesticide/Herbicide Exposure

1. Brief Description of Task (*Insert keywords ONLY such as “vaporisation” or “pouring”, “mix” include type of pesticide if known)*

*_____________________________________________________________________*

*_____________________________________________________________________*

*_____________________________________________________________________*

1. Approximately how many *days* per year involved contact with the pesticide/herbicide?

__________days

1. How much of the *diluted* pesticide was used every time?
2. 1 cup (200ml)
3. 2 to 5 cups (400 to 1000ml)
4. 6 to 9 cups (1200 to 2000ml)
5. 10 cups or more (>2000ml – 2lts)
6. I don’t remember
7. Have you ever been treated by a doctor for poisoning by these chemicals?
8. Yes
9. No

# Section 4Eii: Chemicals/Gases Exposure

1. Brief Description of Task (*Insert keywords ONLY such as “Laboratory – quality assurance” or “Industries - Chemical”, “Research”, include type of chemical/gas if known)*

*_____________________________________________________________________*

*_____________________________________________________________________*

*_____________________________________________________________________*

1. On average how many hours per day involved contact with the chemicals/gases?

__________ hours

1. Approximately how many days per week involved contact with the chemicals/gases?

○ 1 ○ 2 ○ 3 ○ 4 ○ 5 ○ 6 ○ 7

1. How much of the chemical/gases was used per day?

1. Less than 50ml

2. 51 to 500ml

3. 501 – 2000ml

4. More than 2000ml (2lts+)

5. I don’t remember

5. At this work did you ever:

1. Have regular headaches
2. Experience dizzy spells/fainting
3. Become unconscious (excluding head injury)
4. None of the above

# Section 4Fii: Animal Feed Exposure

1. Brief Description of Task (*Insert keywords ONLY such as “Stable Farm Cows” or “Pigs” , “Chicken steroid-enriched food”, include type of food)*

*_____________________________________________________________________*

*_____________________________________________________________________*

*_____________________________________________________________________*

1. On average how many hours per day involved contact with animal feed?

__________ hours

3. Approximately how many days per week involved contact with the animal feed?

○ 1 ○ 2 ○ 3 ○ 4 ○ 5 ○ 6 ○ 7

4. How much of the animal feed was used per day?

1. Less than 15 kg
2. 16 to 30 kg
3. 31 to 50 kg
4. More than 50kg
5. I don’t remember

# Section 4Gii: Cosmetics and Pharmaceuticals Exposure

1. Brief Description of Task (*Insert keywords ONLY such as “mixing” or “vaporisation”)*

*_____________________________________________________________________*

*_____________________________________________________________________*

*_____________________________________________________________________*

1. Approximately how many hours per day involved contact with the product?

__________hours

1. How much of the cosmetic/pharmaceutical product was handled per day? (note: ONLY lab work)
2. Less than 5ml (1 teaspoon eg.)
3. 5 to 10 ml
4. 11 to 20 ml
5. More than 20 ml
6. I don’t remember
7. Not applicable (did not work in Lab)
8. Do you use a microfilter during work?
9. Yes
10. No

# Section 4Biii: Work – Exposure Section

**Please list all the work you have done. At least for 6 months starting from your last regular work.**

*INTERVIEWER: Please specify whether the work actually involved exposure to the substances (eg. A manager of Delimara PS may have had no contact with solvents, etc.)*

**IF ONLY ONE JOB WAS CARRIED OUT FILL IN THIS SECTION ONCE; IF MORE THAN ONE PREVIOUS JOB FILL IN AS MANY AS REQUIRED UP TO THE LAST 4 JOBS**

1. **Job Category (write in) _____________________________________________**

*Here it is up to the interviewer to decide the category that is “most fitting”. For eg. A cleaner would be an “operator” as she would be exposed to cleaning agents, etc.*

1. Administrative
2. Operator
3. Supervisor / middle management
4. Manager
5. Director
6. **FROM** (*year & month) ____________________* **TO** (*year & month) ____________________*
7. **At this workplace was there use of:**
8. Solvents – **ASK SECTION 4Ciii: SOLVENTS EXPOSURE (pg16)**
9. Pesticides/Herbicides – **ASK SECTION 4Diii: PESTICIDES/HERBICIDES (pg17)**
10. Chemical Fumes or Gases – **ASK SECTION 4Eiii: CHEMICAL FUMES/GASES (pg18)**
11. Animal Feed – **ASK SECTION 4Fiii: ANIMAL FEED (pg19)**
12. Pharmaceutical/Cosmetic Products – **ASK SECTION 4Giii: PHARMACEUTICAL/COSMETIC**

**PRODUCTS (pg20)**

1. None of the above - GO TO NEXT SECTION (pg21), LIST THE PREVIOUS

JOB, IF NO OTHER JOB GO TO SECTION 5 (pg27)

# Section 4Ciii: Solvent Exposure

1. Brief Description of Task (*Insert keywords ONLY such as “mixing” or “painting”, include type of solvent if known)*

*_____________________________________________________________________*

*_____________________________________________________________________*

*_____________________________________________________________________*

1. Approximately how many hours per day involved contact with the solvent?

__________ hours

1. Approximately how many days involved contact with the solvent? And how frequent was this?

| Number of days:  _____________________ | Frequency:   1. Weekly 2. Monthly 3. Yearly |
| --- | --- |

1. How much of the solvent/mixture was used per day?
2. ____ spoonful (10ml per spoonful)
3. ____ cup (200ml per cup)
4. I don’t remember
5. At this work did you ever:
6. Have regular headaches
7. Experience dizzy spells/fainting
8. Become unconscious (excluding head injury)
9. None of the above

# Section 4Diii: Pesticide/Herbicide Exposure

1. Brief Description of Task (*Insert keywords ONLY such as “vaporisation” or “pouring”, “mix” include type of pesticides if known)*

*_____________________________________________________________________*

*_____________________________________________________________________*

*_____________________________________________________________________*

1. Approximately how many *days* per year involved contact with the pesticide/herbicide?

__________days

1. How much of the *diluted* pesticide was used every time?
2. 1 cup (200ml)
3. 2 to 5 cups (400 to 1000ml)
4. 6 to 9 cups (1200 to 2000ml)
5. 10 cups or more (>2000ml – 2lts)
6. I don’t remember
7. Have you ever been treated by a doctor for poisoning by these chemicals?
8. Yes
9. No

# Section 4Eiii: Chemicals/Gases Exposure

1. Brief Description of Task (*Insert keywords ONLY such as “Laboratory – quality assurance” or “Industries - Chemical”, “Research” include type of chemical/gas if known)*

*_____________________________________________________________________*

*_____________________________________________________________________*

*_____________________________________________________________________*

1. On average how many hours per day involved contact with the chemicals/gases?

__________ hours

1. Approximately how many days per week involved contact with the chemicals/gases?

○ 1 ○ 2 ○ 3 ○ 4 ○ 5 ○ 6 ○ 7

1. How much of the chemical/gases was used per day?

1. Less than 50ml

2. 51 to 500ml

3. 501 – 2000ml

4. More than 2000ml (2lts+)

5. I don’t remember

5. At this work did you ever:

1. Have regular headaches
2. Experience dizzy spells/fainting
3. Become unconscious (excluding head injury)
4. None of the above

# Section 4Fiii: Animal Feed Exposure

1. Brief Description of Task (*Insert keywords ONLY such as “Stable Farm Cows” or “Pigs” , “Chicken steroid-enriched food”, include type of food)*

*_____________________________________________________________________*

*_____________________________________________________________________*

*_____________________________________________________________________*

1. On average how many hours per day involved contact with animal feed?

__________ hours

3. Approximately how many days per week involved contact with the animal feed?

○ 1 ○ 2 ○ 3 ○ 4 ○ 5 ○ 6 ○ 7

4. How much of the animal feed was used per day?

1. Less than 15 kg
2. 16 to 30 kg
3. 31 to 50 kg
4. More than 50kg
5. I don’t remember

# Section 4Giii: Cosmetics and Pharmaceuticals Exposure

1. Brief Description of Task (*Insert keywords ONLY such as “mixing” or “vaporisation”)*

*_____________________________________________________________________*

*_____________________________________________________________________*

*_____________________________________________________________________*

1. Approximately how many hours per day involved contact with the product?

__________hours

1. How much of the cosmetic/pharmaceutical product was handled per day? (note: ONLY lab work)
2. Less than 5ml (1 teaspoon eg.)
3. 5 to 10 ml
4. 11 to 20 ml
5. More than 20 ml
6. I don’t remember
7. Not applicable (did not work in Lab)
8. Do you use a microfilter during work?
9. Yes
10. No

# Section 4Biv: Work – Exposure Section

**Please list all the work you have done. At least for 6 months starting from your last regular work.**

*INTERVIEWER: Please specify whether the work actually involved exposure to the substances (eg. A manager of Delimara PS may have had no contact with solvents, etc.)*

**IF ONLY ONE JOB WAS CARRIED OUT FILL IN THIS SECTION ONCE; IF MORE THAN ONE PREVIOUS JOB FILL IN AS MANY AS REQUIRED UP TO THE LAST 4 JOBS**

1. **Job Category (write in) _____________________________________________**

*Here it is up to the interviewer to decide the category that is “most fitting”. For eg. A cleaner would be an “operator” as she would be exposed to cleaning agents, etc.*

1. Administrative
2. Operator
3. Supervisor / middle management
4. Manager
5. Director
6. **FROM** (*year & month) ____________________* **TO** (*year & month) ____________________*
7. **At this workplace was there use of:**
8. Solvents – **ASK SECTION 4Civ: SOLVENTS EXPOSURE (pg22)**
9. Pesticides/Herbicides – **ASK SECTION 4Div: PESTICIDES/HERBICIDES (pg23)**
10. Chemical Fumes or Gases – **ASK SECTION 4Eiv: CHEMICAL FUMES/GASES (pg24)**
11. Animal Feed – **ASK SECTION 4Fiv: ANIMAL FEED (pg 25)**
12. Pharmaceutical/Cosmetic Products – **ASK SECTION 4Giv: PHARMACEUTICAL/COSMETIC**

**PRODUCTS (Pg26)**

1. None of the above - GO TO SECTION 5 (pg27)

# Section 4Civ: Solvent Exposure

1. Brief Description of Task (*Insert keywords ONLY such as “mixing” or “painting”, include type of solvent if known)*

*_____________________________________________________________________*

*_____________________________________________________________________*

*_____________________________________________________________________*

1. Approximately how many hours per day involved contact with the solvent?

__________ hours

1. Approximately how many days involved contact with the solvent? And how frequent was this?

| Number of days:  _____________________ | Frequency:   1. Weekly 2. Monthly 3. Yearly |
| --- | --- |

1. How much of the solvent/mixture was used per day?
2. ____ spoonful (10ml per spoonful)
3. ____ cup (200ml per cup)
4. I don’t remember
5. At this work did you ever:
6. Have regular headaches
7. Experience dizzy spells/fainting
8. Become unconscious (excluding head injury)
9. None of the above

# Section 4Div: Pesticide/Herbicide Exposure

1. Brief Description of Task (*Insert keywords ONLY such as “vaporisation” or “pouring”, “mix’, include type of pesticide if known)*

*_____________________________________________________________________*

*_____________________________________________________________________*

*_____________________________________________________________________*

1. Approximately how many *days* per year involved contact with the pesticide/herbicide?

__________days

1. How much of the *diluted* pesticide was used every time?
2. 1 cup (200ml)
3. 2 to 5 cups (400 to 1000ml)
4. 6 to 9 cups (1200 to 2000ml)
5. 10 cups or more (>2000ml – 2lts)
6. I don’t remember
7. Have you ever been treated by a doctor for poisoning by these chemicals?
8. Yes
9. No

# Section 4Eiv: Chemicals/Gases Exposure

1. Brief Description of Task (*Insert keywords ONLY such as “Laboratory – quality assurance” or “Industries - Chemical”, “Research”, include type of chemical /gas if known)*

*_____________________________________________________________________*

*_____________________________________________________________________*

*_____________________________________________________________________*

1. On average how many hours per day involved contact with the chemicals/gases?

__________ hours

1. Approximately how many days per week involved contact with the chemicals/gases?

○ 1 ○ 2 ○ 3 ○ 4 ○ 5 ○ 6 ○ 7

1. How much of the chemical/gases was used per day?

1. Less than 50ml

2. 51 to 500ml

3. 501 – 2000ml

4. More than 2000ml (2lts+)

5. I don’t remember

5. At this work did you ever:

1. Have regular headaches
2. Experience dizzy spells/fainting
3. Become unconscious (excluding head injury)
4. None of the above

# Section 4Fiv: Animal Feed Exposure

1. Brief Description of Task (*Insert keywords ONLY such as “Stable Farm Cows” or “Pigs” , “Chicken steroid-enriched food” include type of food)*

*_____________________________________________________________________*

*_____________________________________________________________________*

*_____________________________________________________________________*

1. On average how many hours per day involved contact with animal feed?

__________ hours

3. Approximately how many days per week involved contact with the animal feed?

○ 1 ○ 2 ○ 3 ○ 4 ○ 5 ○ 6 ○ 7

4. How much of the animal feed was used per day?

1. Less than 15 kg
2. 16 to 30 kg
3. 31 to 50 kg
4. More than 50kg
5. I don’t remember

# Section 4Giv: Cosmetics and Pharmaceuticals Exposure

1. Brief Description of Task (*Insert keywords ONLY such as “mixing” or “vaporisation”)*

*_____________________________________________________________________*

*_____________________________________________________________________*

*_____________________________________________________________________*

1. Approximately how many hours per day involved contact with the product?

__________hours

1. How much of the cosmetic/pharmaceutical product was handled per day? (note: ONLY lab work)
2. Less than 5ml (1 teaspoon eg.)
3. 5 to 10 ml
4. 11 to 20 ml
5. More than 20 ml
6. I don’t remember
7. Not applicable (did not work in Lab)
8. Do you use a microfilter during work?
9. Yes
10. No

# Section 5: Lifestyle Survey

Q21. Do you use Make up or Moisturiser?

1. Yes
2. No

Q22. Make up application area:

1. Mouth
2. Eyes
3. Face
4. Does not apply make up *(for online input leave empty)*

Q23. Moisturiser application area:

1. Face
2. Feet
3. Arms
4. Hands
5. Body
6. Legs
7. Does not apply moisturiser *(for online input leave empty)*

Q24. Have you ever used Tobacco containing products?

1. Yes – **GO TO Q25**
2. No – **GO TO Q26**

Q25. Please provide details of your lifetime tobacco use:

| **FROM** | **To** | **Type of Tobacco** | **Amount** |
| --- | --- | --- | --- |
| *Year* | *Year* | *Choose one category per row* | *Individual units (eg. 1 cigarette, 20g snuff)* |
|  |  | - Cigarette (filter- manufactured) - Cigarette (non-filter) - Cigars - Pipe - Pipe Snuff - Water Pipe (Hookah/Nargilah)   Other (specify) _____________________ |  |
| *Year* | *Year* | *Choose one category per row* | *Individual units (eg. 1 cigarette, 20g snuff)* |
|  |  | - Cigarette (filter- manufactured) - Cigarette (non-filter) - Cigars - Pipe - Pipe Snuff - Water Pipe (Hookah/Nargilah)   Other (specify) _____________________ |  |
| *Year* | *Year* | *Choose one category per row* | *Individual units (eg. 1 cigarette, 20g snuff)* |
|  |  | - Cigarette (filter- manufactured) - Cigarette (non-filter) - Cigars - Pipe - Pipe Snuff - Water Pipe (Hookah/Nargilah)   Other (specify) _____________________ |  |
| *Year* | *Year* | *Choose one category per row* | *Individual units (eg. 1 cigarette, 20g snuff)* |
|  |  | - Cigarette (filter- manufactured) - Cigarette (non-filter) - Cigars - Pipe - Pipe Snuff - Water Pipe (Hookah/Nargilah)   Other (specify) _____________________ |  |

Q26. Are you regularly exposed to at least 1 smoker (home or work)?

1. Yes
2. No

Q27. Have you ever consumed beer, wine or spirits regularly?

*At least one bottle/one glass of wine/ one glass of spirit per week during one year*

1. Yes – **GO TO Q28**
2. No – **GO TO Q29**

Q28. How much Alcohol do you/ did you drink on average per week?

| *Beer (bottles), wine (glasses) or spirits (shots), Prior to Diagnosis if the subject is a case subject.* | | | |
| --- | --- | --- | --- |
| **Years ago** | **Beer (25cl)** | **Wine (200 ml glass)** | **Spirits (Shot glass)** |
| Up to 5 years ago |  |  |  |
| 5-10 years ago |  |  |  |
| 10-20 years ago |  |  |  |
| 20-30 years ago |  |  |  |
| - 1. years ago |  |  |  |

Q29. On average, over the past 10–15 years, how many cups of tea/coffee did you drink per day? *Prior to Diagnosis if the subject is a case subject.*

| **Americano (Nescafe`)** | **Italian Coffee (tal-Magna)** | **Decaf** | **Tea** |
| --- | --- | --- | --- |
|  |  |  |  |

Q30. Do you do physical activity regularly? *Defined as at least 1 hr intentional walking per week*

1. Yes – **GO TO Q31**
2. No – **GO TO Q32**

Q31. Since when do you do regular physical activity? *Up to now*

1. 1 to 2 years
2. 3 to 4 years
3. 5 to 6 years
4. 7 to 8 years
5. 9 to 10 years

Q32. Do you regularly wash vegetables and fruit with water/bicarbonate prior to consumption?

1. Yes
2. No

# Section 6: Food Items

Q33. How often do you consume the following Soya Products?

**SHOW MEASUREMENT PICTURES**

| **Item** | **Servings** | **Frequency** | | | |
| --- | --- | --- | --- | --- | --- |
|  | *Write in number of servings according the given measurement* | *Select frequency below* | | | |
| Soya Dairy Products (Including soya ice-cream) | *1 cup* | ○ Never | ○ Daily | | ○ Monthly |
|  |  | ○ Weekly | | ○ 2-3 times weekly | |
| Tofu (Soy Bean Curd) | *1Wedge* | ○ Never | ○ Daily | | ○ Monthly |
|  |  | ○ Weekly | | ○ 2-3 times weekly | |
| Soya Beans | *1 Handful* | ○ Never | ○ Daily | | ○ Monthly |
|  |  | ○ Weekly | | ○ 2-3 times weekly | |
| Soya meat Substitute | *1 cup* | ○ Never | ○ Daily | | ○ Monthly |
|  |  | ○ Weekly | | ○ 2-3 times weekly | |

Q34. How many portions per week do you consumer of the following?

**SHOW PORTION PICTURES**

| **Item** | **Servings**  *Write in number of servings according the given measurement* | **Frequency** | | | |
| --- | --- | --- | --- | --- | --- |
|  |  | *Select frequency below* | | | |
| Pasta | *1 small bowl* | ○ Never | ○ Daily | | ○ Monthly |
|  |  | ○ Weekly | | ○ 2-3 times weekly | |
| Cereals | *1 small bowl* | ○ Never | ○ Daily | | ○ Monthly |
|  |  | ○ Weekly | | ○ 2-3 times weekly | |
| Sweets (Candy) | *1Handful* | ○ Never | ○ Daily | | ○ Monthly |
|  |  | ○ Weekly | | ○ 2-3 times weekly | |
| Confectionary (Cakes etc.) | *Wedge (1/8^th^ cake slice)* | ○ Never | ○ Daily | | ○ Monthly |
|  |  | ○ Weekly | | ○ 2-3 times weekly | |
| Fruit Juices | *1 Glass (200ml)* | ○ Never | ○ Daily | | ○ Monthly |
|  |  | ○ Weekly | | ○ 2-3 times weekly | |
| Soft Drinks (Fizzy) | *1 Glass (200ml)* | ○ Never | ○ Daily | | ○ Monthly |
|  |  | ○ Weekly | | ○ 2-3 times weekly | |
| Energy Drinks (eg. Gatorade) | *1 Glass (200ml)* | ○ Never | ○ Daily | | ○ Monthly |
|  |  | ○ Weekly | | ○ 2-3 times weekly | |
| Stimulant Drinks (eg. Redbull, Shark) | *1 Glass (200ml)* | ○ Never | ○ Daily | | ○ Monthly |
|  |  | ○ Weekly | | ○ 2-3 times weekly | |

Q35. Do you regularly consume the following?

**SHOW PORTION PICTURES**

| **Item** | **Servings** | **Frequency** | | | |
| --- | --- | --- | --- | --- | --- |
|  | *Write in number of servings according the given measurement* | *Select frequency below* | | | |
| Butter | *1 teaspoon* | ○ Never | ○ Daily | | ○ Monthly |
|  |  | ○ Weekly | | ○ 2-3 times weekly | |
| Lard (includes Pastizzi, cornetti, rosticceria) | *1 teaspoon (pastizzi = 2)* | ○ Never | ○ Daily | | ○ Monthly |
|  |  | ○ Weekly | | ○ 2-3 times weekly | |
| Soft Cheeses | *1 Wedge* | ○ Never | ○ Daily | | ○ Monthly |
|  |  | ○ Weekly | | ○ 2-3 times weekly | |
| Bacon | *1 slice* | ○ Never | ○ Daily | | ○ Monthly |
|  |  | ○ Weekly | | ○ 2-3 times weekly | |
| Mortadella | *1 slice* | ○ Never | ○ Daily | | ○ Monthly |
|  |  | ○ Weekly | | ○ 2-3 times weekly | |
| Salami | *5 small slices* | ○ Never | ○ Daily | | ○ Monthly |
|  |  | ○ Weekly | | ○ 2-3 times weekly | |

Q36. Do you regularly consume the following?

| **Item** | **Servings** | **Frequency** | | | |
| --- | --- | --- | --- | --- | --- |
|  | *Write in number of servings according the given measurement* | *Select frequency below* | | | |
| Tomatoes | *1 medium tomato* | ○ Never | ○ Daily | | ○ Monthly |
|  |  | ○ Weekly | | ○ 2-3 times weekly | |
| Beans | *1 bowl* | ○ Never | ○ Daily | | ○ Monthly |
|  |  | ○ Weekly | | ○ 2-3 times weekly | |
| Carrots | *1 bowl* | ○ Never | ○ Daily | | ○ Monthly |
|  |  | ○ Weekly | | ○ 2-3 times weekly | |
| Cabbage | *1 bowl* | ○ Never | ○ Daily | | ○ Monthly |
|  |  | ○ Weekly | | ○ 2-3 times weekly | |
| Spinach | *1 bowl* | ○ Never | ○ Daily | | ○ Monthly |
|  |  | ○ Weekly | | ○ 2-3 times weekly | |

Q37. Do you regularly consume/use the following products?

| **Item** | **Servings** | **Frequency** | | | |
| --- | --- | --- | --- | --- | --- |
|  | *Write in number of servings according the given measurement* | *Select frequency below* | | | |
| Artificial Sweetener | *1 teaspoon* | ○ Never | ○ Daily | | ○ Monthly |
|  |  | ○ Weekly | | ○ 2-3 times weekly | |
| Low Sodium Salt | *1 teaspoon* | ○ Never | ○ Daily | | ○ Monthly |
|  |  | ○ Weekly | | ○ 2-3 times weekly | |
| Dried Soup Mixes | *1 Bowl* | ○ Never | ○ Daily | | ○ Monthly |
|  |  | ○ Weekly | | ○ 2-3 times weekly | |
| Chinese Food | *1 Bowl* | ○ Never | ○ Daily | | ○ Monthly |
|  |  | ○ Weekly | | ○ 2-3 times weekly | |
| Soy Sauce | *1 teaspoon* | ○ Never | ○ Daily | | ○ Monthly |
|  |  | ○ Weekly | | ○ 2-3 times weekly | |
| Blue Cake Colouring | *1 teaspoon* | ○ Never | ○ Daily | | ○ Monthly |
|  |  | ○ Weekly | | ○ 2-3 times weekly | |
| Soft Drinks (Fizzy) | *1 can 33cl* | ○ Never | ○ Daily | | ○ Monthly |
|  |  | ○ Weekly | | ○ 2-3 times weekly | |
| Crisps (Potato Products) | *Small box (70g)* | ○ Never | ○ Daily | | ○ Monthly |
|  |  | ○ Weekly | | ○ 2-3 times weekly | |
| Processed Meat (usually canned products) | *Can of tinned meat* | ○ Never | ○ Daily | | ○ Monthly |
|  |  | ○ Weekly | | ○ 2-3 times weekly | |
|  |  |  |  | |  |
|  |  |  | |  | |

Q38. Has a doctor ever told you had one of the following illnesses?

1. Diabetes
2. Heart Attack
3. Liver Failure
4. Cushing’s Syndrome
5. Hypothyroidism
6. None of these *(for online input leave empty)*

Q39. Do you use any medication on a daily basis?

1. Metformin (*taz-zokkor*)
2. Diuretics (*tal-pipi … often ending in “-ide”)*
3. Anti-Hypertensives (*tal-pressjoni… often ending in “–tan”)*
4. Calcium Channel Blockers (*tal-pressjoni… often ending in “– dipine”)*
5. ACE inhibitors (*tal-pressjoni/qalb … often ending in “-pril”)*
6. None of these

Q40. Have you ever had radiotherapy/CT scans in the Chest Area?

1. Yes – **GO TO Q41**
2. No - **GO TO Q42**
3. **Had breast cancer**
4. Refuse to answer this question

Q41. If yes, how many times have you had radiotherapy/CT scans in the Chest Area?

__________times

Q42. Between the age of 10 and 29 did you use to spend at least an hour per day exposed to the sun during the summer months (July and September)?

1. Yes **– GO TO Q43**
2. No – **GO TO SECTION 3 (last section, pg 36)**

Q43. If yes, how many hours per day on average?

__________hours

Q44. Did you use sunscreen protection while you were sunbathing?

1. High Factor (50+)
2. Medium Factor (20 – 49)
3. Low Factor (<20)
4. No

# Questionnaire closure

I would like to thank you for your valuable time.

Should you have any questions about this research or about your rights as a participant you can call Professor Christian Scerri on 2340 2268. This number can be found on the information sheet provided.

As a Quality Control Procedure MISCO will be carrying out some call backs to ensure that all work procedure has been correctly followed.  May I please ask you for you contact details?  These contact details will be solely used for quality checking purposes and will not form part of the survey, the answers given by yourself will in no way be linked to you personally.

*Name and Surname: ____________________________________________*

*Telephone or Mobile number: ___________________________________________*
