# Supplementary material for: Environmental and lifestyle risk factors of breast cancer in Malta—a retrospective case-control study
Source: EPMA J. 2016 Sep 20;7(1):20. doi: 10.1186/s13167-016-0069-z (PMC5029064; doi:10.1186/s13167-016-0069-z)
Supplement: Supplementary file 2 — List of variables analysed. (DOCX 14 kb) [file 13167_2016_69_MOESM2_ESM.docx]

Additional file 2 – List of variables analysed

# Physiological Characteristics

- Height (m)
- Weight (kg)
- BMI (kg/m2)
- Menarchal age (years)
- Parity (Yes/No)
- Menopausal age (years)
- Menopausal Status
  - Premenopausal
  - Perimenopausal
  - Postmenopausal
- OCP Use (Yes = 1)
- HRT Use (Yes = 1)

# Lifestyle

- Exercise (Active)
- Exercise (cumulative. hours)
- School leaving age
- Alcohol Status (Yes = 1)
- Alcohol lifetime exposure (units)
- Tobacco (Yes = 1)
- Tobacco lifetime exposure (items)
- Second-hand smoker (Yes =1 )
- Makeup amount (ml/week)
- Moisturiser amount (ml/week)
- Makeup/Moisturiser use (Yes = 1)
- Vegetable Washing (Yes = 1)
- Sunlight exposure age 10-29 (Yes = 1)
- Sun exposure (hrs daily in summer)
- Sunblock Use
  - No use
  - Low Factor
  - Medium Factor
  - High factor

# Work

- Ever Worked (Yes = 1)
- Work Exposure Risk (Yes = 1)

# Diet

- Soya exposure (cups per month)
- Tomatoes (no./month)
- Beans (Cups/month)
- Carrots (Cups/month)
- Cabbage (Cups/month)
- Spinach (Cups/month)
- Artificial Sweetener (tsp/month)
- Low Sodium Salt (tsp/month)
- Dried Soup (cups/month)
- Chinese Food (meals/month)
- Soysauce (tsp/month)
- Softdrink (33cl cans/month)
- Chips (35g packet/month)
- Canned meat (70g tin /month)

# Illness

- Diabetes (Yes = 1)
- Myocardial Episodes (Yes = 1)
- Liver Failure Episodes (Yes = 1)
- Hypothyroidism (Yes = 1)

# Medication

- Metformin (Yes = 1)
- Diuretics (Yes = 1)
- Anti-Hypertensives (Yes = 1)
- ACE Inhibitors (Yes = 1)
- Calcium-channel blockers (Yes = 1)
